# A Porosity Closure Model Under Hot Isostatic Pressing of an IN718 Alloy Manufactured by Powder Bed Fusion

**DOI:** 10.3390/ma18051001

**Published:** 2025-02-24

**Authors:** Xuming Wang, Liqun Niu, Kaixiong Cheng, Bingzheng Wang, Qi Zhang

**Affiliations:** 1 Lanzhou Lanshi Super Alloy New Material Co., Ltd., Lanzhou 730000, China; wxm0720@163.com (X.W.); abing69428@163.com (B.W.); 2 School of Mechanical and Electronical Engineering, Lanzhou University of Technology, Lanzhou 730050, China; 13993207781@163.com; 3 School of Mechanical Engineering, Xi’an Jiaotong University, Xi’an 710049, China; henryzhang@mail.xjtu.edu.cn

**Keywords:** powder bed fusion, hot isostatic pressing, porosity closure model, IN718, molecular dynamics simulation

## Abstract

The low productivity and high cost of additive manufacturing techniques, such as powder bed fusion (PBF), limits its wide application in industry. A combined approach of hot isostatic pressing (HIP) and PBF was an effective means to solve this limitation. Nevertheless, there is currently a lack of a porosity closure model to design and optimize the HIP process parameters of PBF-manufactured components. The porosity closure condition of the PBF-manufactured component is deduced based on the additivity of logarithmic strain and the plastic equation of volume compressible material, and then a porosity closure model considering temperature and pressure is established and verified by molecular dynamics simulation. Subsequently, a HIP diagram of the PBF-manufactured IN718 is constructed. Four different initial relative densities of 0.956, 0.970, 0.984, and 0.996 of IN718 components are introduced by increasing the scanning speed of PBF. HIP post-treatment experiments of different relative density components are performed. The accuracy of the HIP diagram is verified by the relative density test and microstructure observation.

## 1. Introduction

Additive manufacturing (AM) has shown great potential and increasing interest in complicated structure components because it is a bottom-up manufacturing method contrary to conventional technologies [1,2]. For instance, powder bed fusion (PBF) has been widely used in various fields such as aerospace, aircraft, and medical implants [3,4]. However, low productivity and high cost are major barriers to the widespread application of PBF. The high-speed parameters (high scan speed, wide layer thickness, or big hatch distance) can improve productivity while introducing porosities that lack fusion, worsening the performance of the component. To address this issue, a post-processing treatment, such as hot isostatic pressing (HIP), is a practical and effective means [5,6]. Therefore, Herzog et al. [7] pointed out that a combined approach of HIP and PBF was an effective means to improve productivity and reduce cost.

Currently, the effect of HIP on the porosity of AM components has been extensively studied by many researchers. Qiu et al. [8] studied the effect of HIP on the porosity and performance of Ti6Al4V manufactured by PBF. The results showed that almost all of the porosity defects were closed after HIP under 920 °C and 103 MPa for a duration of 4 h, which ameliorated ductility. Chen et al. [9] presented the effect of HIP on the porosity of Ti6Al4V manufactured by cold spray AM. The results showed that the interior porosity defects could be eliminated through HIP at 920 °C and 120 MPa for a duration of 2 h. Tillmann et al. [6] studied the influence of the HIP process parameters on the porosity of PBF-manufactured IN718 components by means of micro-X-ray computed tomography (XμCT). The results showed that the effect of temperature on porosity closure is greater than pressure. Liverani et al. [10] investigated the influence of different pressures on the porosity of 316L by PBF under 1150 °C and a duration of 3 h. The results showed that the pressure of 5 MPa has no effect on elimination porosity, but 100–150 MPa can achieve a good effect. However, the abovementioned investigations mainly focused on physical experiments and did not quantitatively characterize the relationship between porosity and temperature/pressure during HIP.

In the industry, XμCT and ultrasonic were often used to check the porosity of AM products. However, the XμCT analyses were typically expensive and not possibly realized on full-scale testing, and microscale porosities were undetectable due to resolution limits. Tammas-Williams et al. [11] used HIP to close the porosity of Ti6Al4V manufactured by AM and found that microscale porosity (<5 μm) was undetectable by XμCT. Ultrasonic testing was another way to detect porosities, but its accuracy was very low for AM products [12]. Therefore, the prediction of porosity by numerical methods or mathematical models was of great significance for HIP process design and optimization.

The representative methods in simulating the HIP process involved the finite element method (FEM), discrete element method (DEM), multi-particle finite element method (MPFEM), and molecular dynamics method (MDM). For example, Jinka [13] used the FEM to predict the densification process during the HIP of nickel-based alloy powder metallurgy (PM). Kohar et al. [14] employed the constitutive model developed by Van Nguyen et al. [15] to present an integration scheme that accelerates FEM simulations during the HIP of PM. In addition, many researchers [16,17] used different constitutive models to simulate the powder compaction process during HIP and obtained good predictions. However, the FEM mainly focused on the macroscopic performance under the premise of continuum models and ignored the heterogeneous micro-porosity defects. For the DEM, a typical limitation was that it could not simulate HIP under high relative density (ρ), especially near full density [18,19]. Harthong et al. [19] proposed a meshed DEM, which can increase the simulated ρ from 0.8 to approximately 0.95. Even so, the DEM cannot be used to simulate the HIP process of AM because the ρ is generally greater than 0.95. Procopio and Zavaliangos [20] developed the MPFEM by combining the characteristics of the DEM and FEM to simulate the compaction of particulate material. Li et al. [21] used the MPFEM to simulate the HIP of Ti6Al4V powders and studied the influence of capsule shape, temperature, pressure, and powder type on the densification behavior. However, the objects of the above methods are the HIP process of PM, without the HIP of AM. Moreover, these methods cannot simulate the evolution of micro-porosity defects. The MDM was a powerful method used to simulate the evolution of micro-porosity defects during the AM post-processing treatment HIP process. Recently, Kurian and Mirzaeifar [22] used the MDM to observe the evolution of the porosity in integrated the μ-SLM and HIP processes, but they did not analyze the relationship between porosity and temperature/pressure in the HIP process.

Numerous mathematical models have been defined for optimizing HIP parameters in the literature. Some scholars [23,24] proposed the porosity closure models through the PM theory and others [25,26] also derived some analytical solutions based on a continuum theory. In addition, Zhou et al. [27] proposed a porosity healing model under HIP based on the healing characteristics and thermodynamic theory. However, these models were limited in industrial applications due to complex mathematical forms. Therefore, the purpose of this work was to propose a simple and effective porosity closure model for the AM post-processing treatment HIP process.

From the microscale viewpoint, we believed that PBF-manufactured components included abundant microscale porosities. Both the MDM and the theoretical analysis combined to establish a porosity closure model for the AM post-processing treatment HIP process in this work. Firstly, we used the physical equation (PE) of the volume compressible material (VCM) to analyze the porosity closure condition. Secondly, the MDM was applied to study the relationship between temperature and yield strength and define and verify a porosity closure model considering temperature and pressure. Finally, we constructed a HIP diagram based on temperature and pressure and verified its effectiveness through the HIP experiment of PBF-manufactured IN718 components.

## 2. Deformation Analysis and PE

The formation of microscopic porosity in the AM process is unavoidable. The purpose of AM post-processing treatment HIP is to eliminate porosity defects. In the HIP process, we believe that the component property produced by AM is similar to PM, and the volume changes in deformation. This section uses the additivity of logarithmic deformation and the PE of VCM to derive the porosity closure condition.

### 2.1. Deformation Analysis

Due to the volume changes during HIP, logarithmic strain is used to analyze the deformation.

(1) $$d\varepsilon =\frac{dl_{i}}{l_{i}},\;(i=1,2,3)$$

where dε is logarithmic strain, $dl_{i}$ is the instantaneous unit body length increment, and $l_{i}$ is the instantaneous unit body length.

Then, the unit body equivalent strain increment $d\varepsilon_{e}$ and volume strain increment dθ are defined by Equation (2) as follows:

(2) $$\left\{\begin{array}{c} d\varepsilon_{e}=\sqrt{\frac{2}{9}\left({\left(d\varepsilon_{11}-d\varepsilon_{22}\right)}^{2}+{\left(d\varepsilon_{22}-d\varepsilon_{33}\right)}^{2}+{\left(d\varepsilon_{33}-d\varepsilon_{11}\right)}^{2}\right)}\\ d\theta =d\varepsilon_{11}+d\varepsilon_{22}+d\varepsilon_{33} \end{array}\right.$$

Assuming the volume of the unit body is V, the volume of the inner porosity is $V_{void}$, and the porosity volume fraction f can be calculated using Equation (3) as follows:

(3) $$f=\frac{V_{void}}{V}$$

In the plastic deformation analysis of VCM, the conversion relationship of ρ and f is as follows:

(4) $$\left\{\begin{array}{c} \rho =\frac{m_{s}}{m_{l}}\\ m_{l}=\rho_{l}V\\ m_{s}=\rho_{l}\left(V-V_{void}\right) \end{array}\right.$$

where ρ, $\rho_{l}$, $m_{l}$, and $m_{s}$ represent relative density, theoretical density, theoretical mass, and practical mass, respectively.

Bring Equation (3) into Equation (4) to obtain the following:

(5) $$\left\{\begin{array}{c} m_{s}=\rho_{l}\left(1-f\right)V\\ \rho =1-f \end{array}\right.$$

Next, the relationship between dθ and df is deduced. The variation of f at each increment step is defined using the following formula:

(6) $$\left\{\begin{array}{c} df=f_{1}-f_{0}\\ df=\frac{V_{void1}}{V_{1}}-\frac{V_{void0}}{V_{0}} \end{array}\right.$$

where $f_{0}$, $V_{void0}$, and $V_{0}$ represent porosity volume fraction, porosity volume, and unit body volume before deformation, respectively. $f_{1}$, $V_{void1}$, and $V_{1}$ represent parameters after deformation, respectively.

The variation of volume strain dθ at each increment step is calculated by Equation (7).

(7) $$d\theta =\frac{V_{1}-V_{0}}{V_{0}}=\frac{dV}{V_{0}}$$

Because the solid material volume is constant during deformation, only the porosity volume changes, so there is the following:

(8) $$\left\{\begin{array}{c} V_{1}=V_{0}+dV\\ V_{void1}=V_{void0}+dV \end{array}\right.$$

Equation (9) can be deduced by Equations (6) and (8), which are combined as follows:

(9) $$df=\frac{V_{void0}+dV}{V_{0}+dV}-\frac{V_{void0}}{V_{0}}=\left(\frac{V_{0}-V_{void0}}{V_{0}+dV}\right)\left(\frac{dV}{V_{0}}\right)$$

Due to dV ≪ $V_{0}$, Equation (9) is expressed by Equation (10).

(10) $$df=\left(\frac{V_{0}-V_{void0}}{V_{0}}\right)\left(\frac{dV}{V_{0}}\right)$$

Bring Equations (3) and (7) into Equation (10), and the relationship between dθ and df can be written as follows:

(11) $$df=\left(1-f\right)d\theta$$

### 2.2. PE of VCM

The yield condition [28] and flow equation [29] of the VCM are expressed using the following formula:

(12) $$\left\{\begin{array}{c} AJ_{2}+BI_{1}^{2}=CY_{0}^{2}\\ d\varepsilon_{ij}=d\lambda \left(A\sigma_{ij}^{\prime }+2BI_{1}\delta_{ij}\right)\\ I_{1}=\delta_{ij}\sigma_{ij}=3\sigma_{kk}=\sigma_{11}+\sigma_{22}+\sigma_{33}\\ J_{2}=\frac{1}{2}\sigma_{ij}^{\prime }\sigma_{ij}^{\prime }=\frac{1}{6}\left[{\left(\sigma_{11}^{\prime }-\sigma_{22}^{\prime }\right)}^{2}+{\left(\sigma_{22}^{\prime }-\sigma_{33}^{\prime }\right)}^{2}+{\left(\sigma_{33}^{\prime }-\sigma_{11}^{\prime }\right)}^{2}\right] \end{array}\right.$$

where $I_{1}$ represents the first invariant; $J_{2}$ represents the second deviatoric invariant; $Y_{0}$ represents the yield stress of material without porosity; $d\varepsilon_{ij}$ and dλ are the strain increment and scale factor, respectively; $\sigma_{ij}$ and $\sigma_{ij}^{\prime }$ are the stress tensor and stress deviator, respectively; *A*, *B*, and *C* are material parameters; and $\delta_{ij}$ is the Kronecker Delta (*i, j* = 1, 2, 3).

### 2.3. Porosity Closure Condition

In the deformation process, the unit body should satisfy Equation (12). The flow equation can be written as Equation (13).

(13) $$\left\{\begin{array}{c} d\varepsilon_{11}=d\lambda \left(A\sigma_{11}^{\prime }+2BI_{1}\right)\\ d\varepsilon_{22}=d\lambda \left(A\sigma_{22}^{\prime }+2BI_{1}\right)\\ d\varepsilon_{33}=d\lambda \left(A\sigma_{33}^{\prime }+2BI_{1}\right) \end{array}\right.$$

Equation (14) can be derived by combining Equations (2) and (13).

(14) $$d\theta =d\lambda A\left(\sigma_{11}^{\prime }+\sigma_{22}^{\prime }+\sigma_{33}^{\prime }\right)+6d\lambda BI_{1}$$

According to the definition of the first deviatoric invariant, $J_{1}=\sigma_{11}^{\prime }+\sigma_{22}^{\prime }+\sigma_{33}^{\prime }=0$, we obtain the following:

(15) $$d\theta =6d\lambda BI_{1}$$

The general form of Equation (13) can be transformed into Equation (16).

(16) $$\left\{\mathrm{equation}\begin{array}{c} d\varepsilon_{11}-d\varepsilon_{22}=d\lambda A\left(\sigma_{11}^{\prime }-\sigma_{22}^{\prime }\right)\\ d\varepsilon_{22}-d\varepsilon_{33}=d\lambda A\left(\sigma_{22}^{\prime }-\sigma_{33}^{\prime }\right)\\ d\varepsilon_{33}-d\varepsilon_{11}=d\lambda A\left(\sigma_{33}^{\prime }-\sigma_{11}^{\prime }\right) \end{array}\right.$$

Substitute the above into Equation (2) and obtain

(17) $$d\varepsilon_{e}=\frac{2\sqrt{3}}{3}d\lambda A\sqrt{J_{2}}$$

Equation (18) can be derived by combining Equations (15) and (17).

(18) $$d\theta =\frac{3\sqrt{3}BI_{1}}{A\sqrt{J_{2}}}d\varepsilon_{e}$$

Substitute the yield condition $AJ_{2}+BI_{1}^{2}=CY_{0}^{2}$ into Equation (18), which gives

(19) $$Y_{0}\sqrt{AC}\sqrt{1-\frac{BI_{1}^{2}}{CY_{0}^{2}}}d\theta =3\sqrt{3}BI_{1}d\varepsilon_{e}$$

Note that Equation (19) has a real solution, $\frac{BI_{1}^{2}}{CY_{0}^{2}}\leq 1$. $\sqrt{1-\frac{BI_{1}^{2}}{CY_{0}^{2}}}$ satisfies the convergence condition of the power series, which is expressed as $\sqrt{1-\frac{BI_{1}^{2}}{CY_{0}^{2}}}=1-\frac{B}{2C}\frac{I_{1}^{2}}{Y_{0}^{2}}$.

Hence, Equation (19) can be written as follows:

(20) $$Y_{0}\sqrt{AC}\left[1-\frac{B}{2C}{\left(\frac{I_{1}}{Y_{0}}\right)}^{2}\right]d\theta =3\sqrt{3}BI_{1}d\varepsilon_{e}$$

Based on the yield condition $\left(A=3,B=\frac{1}{4{\left[\ln\left(1-\rho \right)\right]}^{2}},C=1\right)$ proposed by Green [29] and Equation (11), Equation (20) becomes the following:

(21) $$\frac{8Y_{0}{\left(\ln(f)\right)}^{2}}{1-f}df-\frac{I_{1}^{2}}{Y_{0}}\frac{df}{\left(1-f\right)}=6I_{1}d\varepsilon_{e}$$

Since the f in the AM process is much less than 1, Equation (22) is obtained as follows:

(22) $$8Y_{0}\int_{f_{1}}^{f_{2}}\frac{{\left(\ln f\right)}^{2}}{1-f}df=8Y_{0}\int_{f_{1}}^{f_{2}}{\left(\ln f\right)}^{2}df$$

Under the assumption that $\varphi \left(f\right)=f\left[{\left(\ln f\right)}^{2}-2\ln f+2\right]$, then

(23) $$8Y_{0}\int_{f_{1}}^{f_{2}}\frac{{\left(\ln f\right)}^{2}}{1-f}df=8Y_{0}\left[\varphi (f_{2})-\varphi (f_{1})\right]$$

where $f_{1}$ represents the porosity volume fraction before HIP and $f_{2}$ represents the porosity volume fraction after HIP.

The second term on the left in Equation (21) changes from the second mean theorem of integration to the following:

(24) $$\frac{1}{Y_{0}}\int_{f_{1}}^{f_{2}}\frac{I_{1}^{2}df}{1-f}=\frac{I_{1}^{2}(f_{g})}{Y_{0}}\ln\frac{1-f_{1}}{1-f_{2}}$$

where $f_{2}\leq f_{g}\leq f_{1}$.

By combining Equations (22)–(24), Equation (21) becomes the following:

(25) $$8Y_{0}\left[\varphi (f_{2})-\varphi (f_{1})\right]-\frac{I_{1}^{2}(f_{g})}{Y_{0}}\ln\frac{1-f_{1}}{1-f_{2}}=6I_{1}\varepsilon_{e}$$

When the porosities are compacted, $f_{2}=0$ and $\underset{f_{2}\to 0}{\lim}\varphi \left(f_{2}\right)=0$, Equation (25) becomes the following:

(26) $$8Y_{0}\left[-\varphi (f_{1})\right]-\frac{I_{1}^{2}(f_{g})}{Y_{0}}\ln(1-f_{1})=6I_{1}\varepsilon_{e}$$

For the HIP process, $\sigma_{11}=\sigma_{22}=\sigma_{33}$. Equation (27) can be obtained as follows:

(27) $$\left\{\begin{array}{c} I_{1}=3\sigma_{m}\\ \varepsilon_{e}=0 \end{array}\right.$$

The porosity closure condition can be directly obtained by combining Equation (26) and Equation (27).

(28) $$\frac{-\varphi (f)}{\ln(1-f)}=\frac{-f\left[{\left(\ln f\right)}^{2}-2\ln f+2\right]}{\ln(1-f)}=\frac{9\sigma_{m}^{2}}{8Y_{0}^{2}}$$

where $I_{1}\left(f_{g}\right)$ is the largest in the HIP process and is represented by $I_{1}$, and $\sigma_{m}$ represents hydrostatic pressure.

Equation (28) is the porosity closure condition, and it shows that the porosity volume fraction (f) is not only related to hydrostatic pressure ($\sigma_{m}$) but also to yield strength without porosity ($Y_{0}$).

## 3. Development of a Porosity Closure Model

### 3.1. Molecular Dynamics Simulation

**Figure 1:**
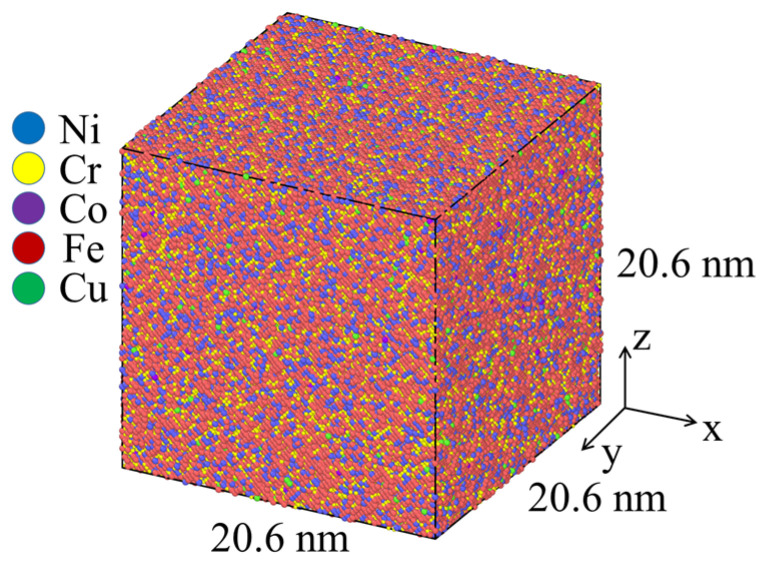
Simulation sample model of the IN718 alloy.

The Large-scale Atomic/Molecular Massively Parallel Simulator (LAMMPS. 2 Aug 2023) [30] was performed to study the relationship between temperature and yield strength for IN718 without porosity. The atomic number of each element is shown in Table 1, a total of 740,772 atoms. The simulation sample is shown in Figure 1, and its size is 20.6 × 20.6 × 20.6 nm^3^. To make the simulation accurate, each atom was randomly distributed.

In order to reduce the size effect that the number of atoms in the simulated system was smaller than that in the real system, the periodic boundary condition was applied in the x, y, and z directions. Prior to the tensile simulation, all samples were first relaxed by 100 ps at isothermal isobaric ensemble (NPT) and preset temperatures to eliminate the influence of the irrational structure in the simulated system. The preset temperatures were 1300 K, 1350 K, 1400 K, 1450 K, and 1500 K, respectively, and the time step was 0.1 × 10^9^ s. Subsequently, the samples were compressed at 5 × 10^9^ s^−1^ until the strain reached 0.3 along the X-axis; the NPT was used, and the other two directions were maintained at 0 GPa. In this work, the EAM potential function proposed by Farkas for FCC structure five-element alloys was used to describe the interaction of Ni, Cr, Co, Fe, and Cu elements [31] and because it has been widely used in the deformation behavior simulations by many researchers [32,33].

### 3.2. Establishment of the Porosity Closure Model

**Figure 2:**
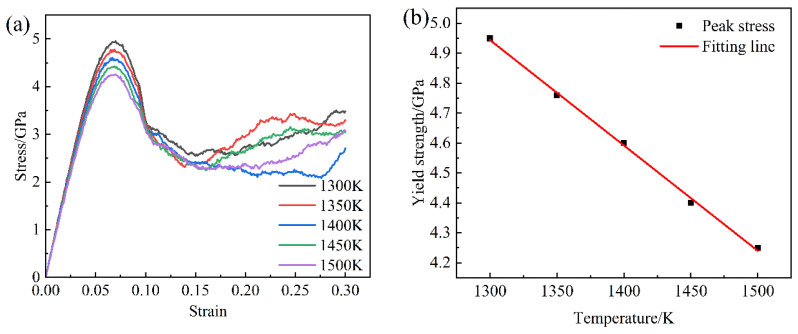
(**a**) Stress–strain curves at different temperatures, (**b**) relationship of temperature and yield strength.

The stress–strain curves at varying temperatures are presented in Figure 2a. It can be observed in the figure that the peak stress increases with the increase in temperature. The peak stress is defined as the yield stress. Hence, the relationship between temperature (*T*) and yield strength (*Y*_0_) is shown in Figure 2b, and that can be fitted very well by Equation (29) as follows:

(29) $$Y_{0}=D+ET$$

where *D* and *E* are material constants.

Hence, the porosity closure model of HIP may be defined as a function of $\sigma_{m}$ and *T* by Equations (28) and (29) as follows:

(30) $$\frac{-f\left[{\left(\ln f\right)}^{2}-2\ln f+2\right]}{\ln(1-f)}=\frac{(\rho -1)\left[{\left(\ln(1-\rho )\right)}^{2}-2\ln(1-\rho )+2\right]}{\ln(\rho )}=\frac{9\sigma_{m}^{2}}{8{\left(D+ET\right)}^{2}}$$

where $\sigma_{m}$ is the pressure in HIP.

However, the current porosity closure model has limitations, such as being unsuitable for printing complex shapes. This is because the closure and growth of porosity are often closely related to the stress state [34]. The printing of complex shapes causes local variations in the stress state (stress triaxiality and Lode parameter). In future research, the model can incorporate the effects of stress state, combined with FEM or machine learning methods, to enable its application to printing complex shapes.

### 3.3. MDM Verification

**Figure 3:**
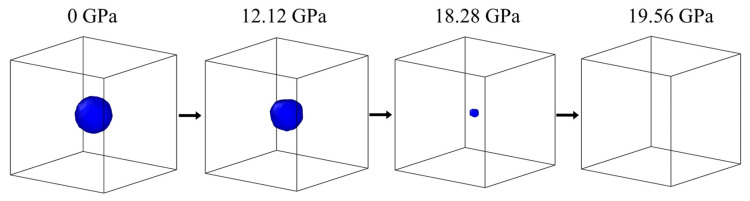
Pressurization stages of a simulation sample with a relative density of 0.956 at 1300 K.

To verify the porosity closure model of HIP (Equation (30)), the sample model of a spherical void was modeled, as shown in Figure 3. The spherical void was located in the center of the simulated sample. The relative density (0.956, 0.970, and 0.984) of the simulated sample was changed by adjusting the radius. The simulation samples with different relative densities were heated to the specified temperature (1300 K, 1400 K, and 1500 K) at 0 GPa, the isothermal relaxation was 20 ps with 200,000 steps, and the time step was 0.1 × 10^9^ s, and then the compressed triaxle was conducted at 5 × 10^9^ s^−1^ until the press reached the target value. The specific simulation parameters are shown in Table 2. The simulation data analysis and visualization were carried out by the Open OVITO code [35]. Importantly, the voids were found to be closed by constructing surface mesh at the conditions in Table 2. For example, Figure 3 shows the porosity evolution process of the simulation sample with a relative density of 0.956 under pressure from 0 GPa to 19.56 GPa at 1300 K.

**Figure 4:**
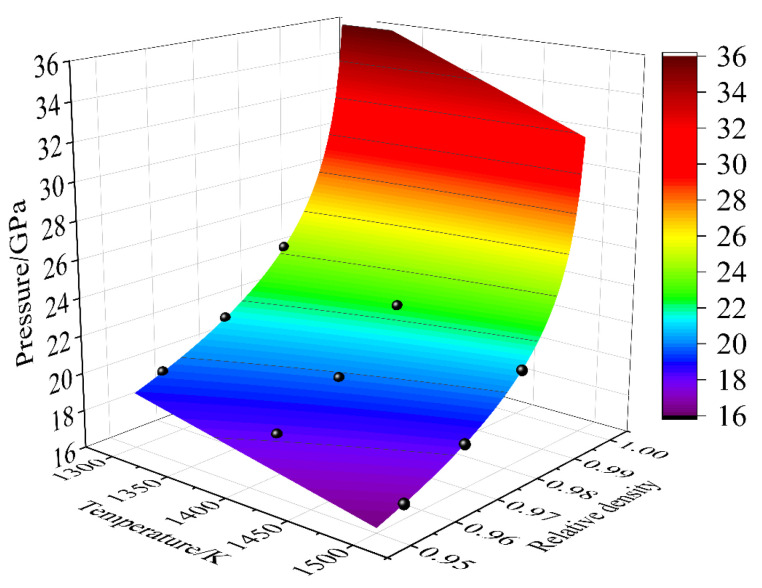
The HIP diagram predicted by the porosity closure model considering the temperature and press for the IN178 alloy.

D and E were obtained by means of the regression analysis, giving *D* = 9.52 and *E* = −0.00352 in Figure 2b. Hence, the HIP diagram was constructed, as displayed in Figure 4. Obviously, the MDM results well agreed with the HIP diagrams. Meanwhile, the target pressure of void closure decreased with the increase in temperature under a certain relative density, as shown in Figure 4. This was consistent with the view of reference [21]. The results showed that the porosity closure model had good predictability for the IN718 alloy under the HIP simulation process.

## 4. Experimental Procedure

### 4.1. PBF Processing Parameters

The nickel alloy IN718 powder was used and was supplied by the Ningbo Zhongyuan new material Technology Ltd., P.R. China (Ningbo, 315000, China). To accurately characterize the powder layer quality, we employed a laser diffraction particle size analyzer (Malvern Mastersizer 3000, Malvern Panalytical, Malvern, UK), which utilizes laser diffraction to measure the particle size distribution. The instrument covers a size range from a few microns to several hundred microns. The gas-atomized spherical powder particle size distribution ranged from 15 to 45 μm (D10–D90) in this study. This is because the powder size distribution is closely correlated with both powder layer quality and defect formation [36]. However, this article does not address this factor at this stage.

**Figure 5:**
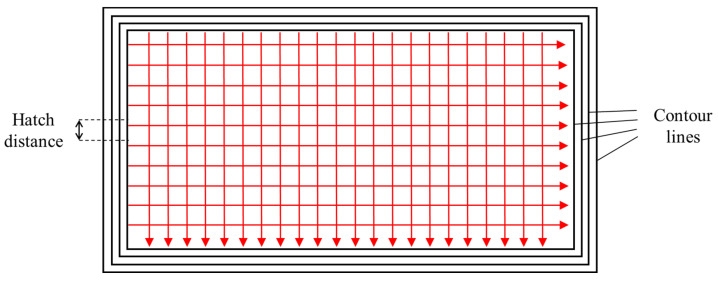
Single-layer scan strategy of a block sample.

All samples were fabricated by the laser PBF technology using a Concept Laser M2 machine (GE Company, Boston, MA, USA) in an argon atmosphere. The manufacturing parameters are presented in Table 3. The laser power was 160 W, the laser spot diameter was 130 μm, the powder thickness was 50 μm, and the hatch distance was 100 μm. Increased porosity fraction was produced by the amplification of the scanning speed (SS). The SS was 500, 650, 850, and 1100 mm/s, respectively. To meet the requirements of subsequent HIP experiments, four contour lines were applied at the lower scanning speed of 500 mm/s to obtain a dense shell. The different SS was only applied to the core of the samples, as shown in Figure 5. The dimension of PBF cubical samples is 15 mm × 20 mm × 35 mm.

### 4.2. HIP Processing Parameters

In this work, the influence of holding time was ignored, and the influence of temperature and pressure on density was mainly studied. According to previous studies of HIP experiment parameters for AM IN718, the longest holding time was 240 min [37,38]. Therefore, the holding time was uniformly used for 240 min. When the holding time was reached, the samples were cooled in a furnace at a preset pressure. To verify the accuracy of the porosity closure model, eight different HIP processing parameters were applied using RD1200 (Quintus Technologies, Västerås, Sweden), as shown in Table 4.

## 5. Results and Discussion

### 5.1. Porosity Analysis

**Figure 6:**
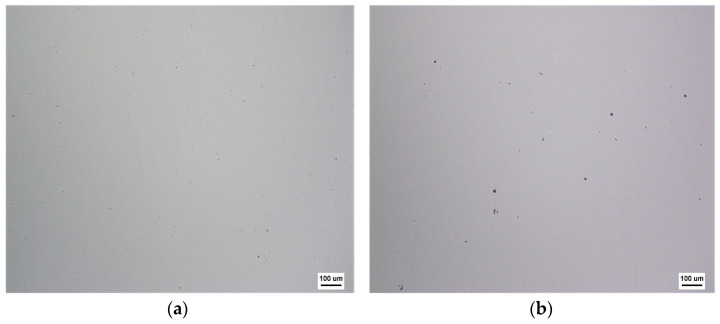

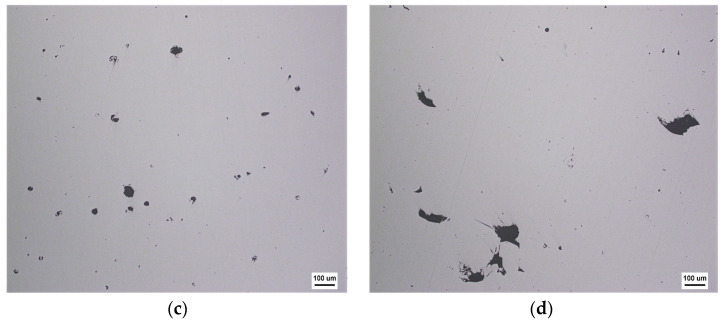
Optical microscope images of the PBF-manufactured IN718 before HIP. (**a**) Case (a-3); (**b**) Case (b-6); (**c**) Case (c-9); (**d**) Case (d-12).

PBF-manufactured samples were divided along the parallel printing direction, which was ground and polished. The microstructure was observed by a Leica Dmi5000m optical microscope. The etching solution was a C_2_H_5_OH (100 mL) + HCl (100 mL) + CuCl_2_ (5 g) mixed solution. Figure 6 shows the metallographic structure of the PBF sample (the relative density is 0.970), and the typical porosities can be seen. The SS of Case (a-3), Case (b-6), Case (c-9), and Case (d-12) in the figure was 500, 650, 850, and 1100 mm/s, respectively. It can be seen that the porosity content of the sample with a varied SS was different. The high SS improved the productivity of PBF, and it also resulted in an increase in sample porosity. This phenomenon can be attributed to the influence of the SS on the heat input and the resulting volumetric energy density during the laser scan [39,40]. The volumetric energy density *E*_*v*_ is given by the ratio of the laser power *P* to the scan speed *v*, laser spot size *d*, and layer thickness *h*.

(31) $$E_{v}=\frac{p}{v\times d\times h}$$

A higher scanning speed reduces the amount of energy deposited per unit volume, leading to lower fusion quality. This insufficient heat input may result in a lack of fusion porosity, where regions of the material fail to fuse properly during the scanning process. Furthermore, excessive scanning speeds can cause keyhole porosity, where the laser power is not sufficient to maintain a stable melt pool, leading to deep, narrow voids that do not close properly. In summary, while increasing the SS can accelerate the PBF process, it must be carefully controlled to avoid detrimental effects on material quality.

The density of samples with different porosity fractions was tested by the MH-100E electron densitometer. The density measured directly by the densitometer was the average of the porous core region and the dense shell. Hence, the density of the core of the sample was calculated by following Equation (31).

(32) $$\rho_{core}=\frac{\rho_{a}V_{l}-\rho_{shell}V_{shell}}{V_{core}}$$

where $\rho_{core}$ is the density of the core of the sample, $\rho_{shell}$ is the density of the dense shell of the sample, $\rho_{a}$ is the measured density of the sample, $\rho_{l}$ is theoretical density, $V_{core}$ is the volume of the core of the sample, $V_{shell}$ is the volume of the dense shell of the sample, and $V_{l}$ is theoretical volume.

The theoretical density of IN718 without porosity is 8.22 gcm^−3^. The shell of the sample is assumed to be dense, and $\rho_{l}$ = $\rho_{shell}$ is 8.22 gcm^−3^. According to the previous PBF processing parameters (Figure 5), it can be seen that $V_{l}$, $V_{shell}$, and $V_{core}$ are equal to 10.5, 1.175712, and 9.324288 cm^3^, respectively. It can be observed in Table 5 that the relative density of the PBF-manufactured IN718 after HIP was higher than that before HIP. Even the highest temperature (1150 °C) and pressure (180 MPa) cannot make the relative density equal to 1. The pressure of HIP increased rapidly with the increase in relative density, as shown in Figure 4. So, when the relative density was close to 1, the required pressure for porosity closure was high, and the equipment capacity was difficult to meet the demand. Therefore, if the relative density is equal to 0.999, the HIP experiments were considered successful and were denoted as $\rho_{amp}=1$.

### 5.2. Construction of the HIP Diagram

**Figure 7:**
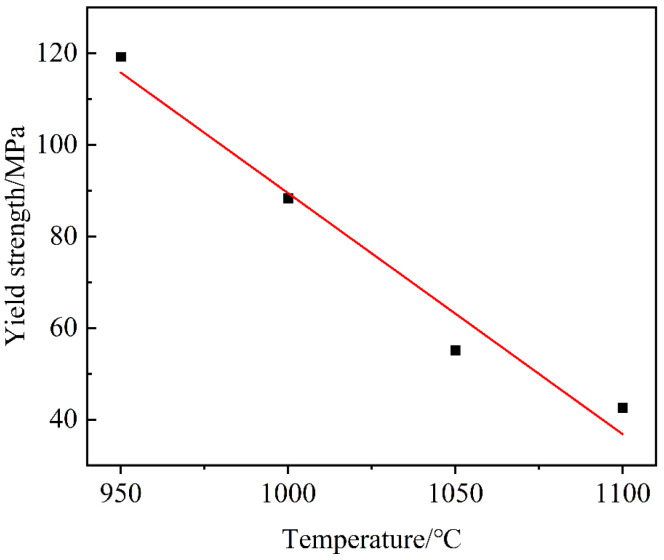
The relationship of the temperature and yield strength of the wrought IN718 alloy.

To construct the HIP diagram of the PBF-manufactured IN718, the yield strength and temperature relationship of IN178 without porosity were needed. So, the experimental data of the wrought IN718 alloy was used and reported by Azarbarmas et al. [41]. The yield strength and temperature relationship of the wrought IN718 at a strain rate of 0.001 s^−1^ is shown in Figure 7.

**Figure 8:**
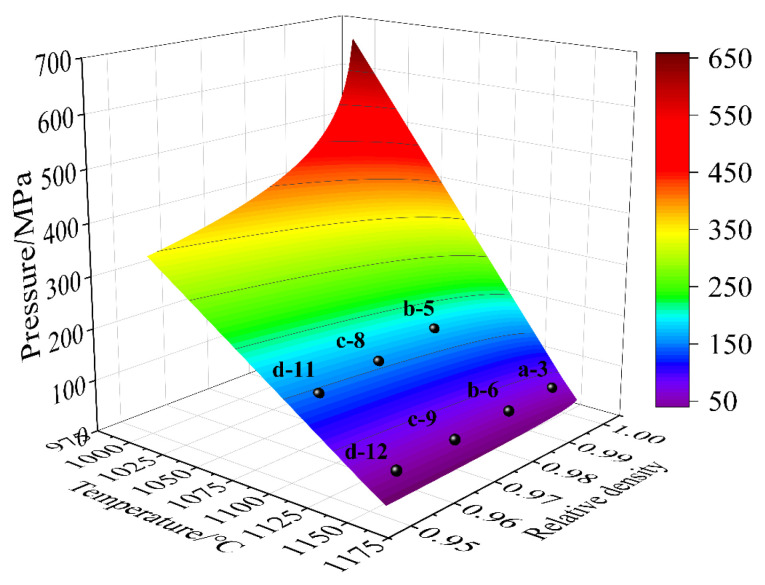
The HIP diagram predicted by the porosity closure model considering the temperature and press for the PBF-manufactured IN718 alloy.

**Figure 9:**
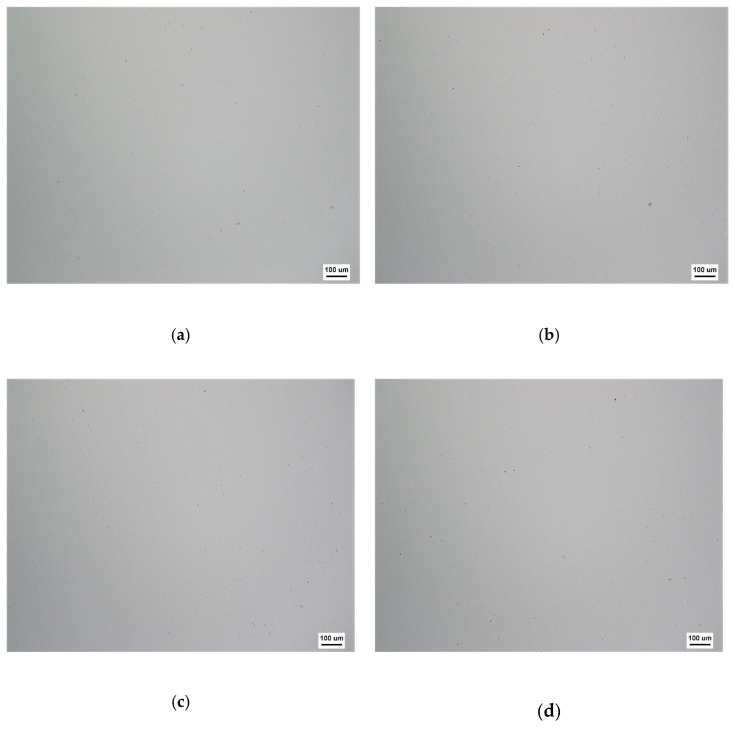
Optical microscope images of the PBF-manufactured IN718 alloy after HIP: (**a**) Case (a-3); (**b**) Case (b-6); (**c**) Case (c-9); (**d**) Case (d-12).

D and E were obtained by means of the regression analysis, giving *D* = 615.846 and *E* = −0.526 in Figure 7. Hence, the HIP diagram of the PBF-manufactured IN718 was constructed, as displayed in Figure 8. Obviously, the results of Cases (a-3, b-5, b-6, c-8, c-9, d-11, and d-12) well agreed with the HIP diagrams. However, other Cases (a-1, a-2, b-4, c-7, and d-10) did not reach full density ($\rho_{amp}=1$). This was because the HIP process parameters did not meet the porosity closure model (Equation (28)). For example, the optimal pressure for Case (d-10) was 250 MPa in the HIP diagram. However, the experimental pressure was 180 MPa, which was less than 250 MPa, so the relative density was 0.961 < 0.999. At the same time, porosities cannot be observed in optical microscope images of the PBF-manufactured IN718 after HIP, as shown in Figure 9. This is mainly due to the following two reasons: (1) pressure-driven closure of porosities. Under HIP conditions, the material is subjected to external pressure, which acts in all directions of the porosities, pushing the gas inside the porosities out and thus promoting the closure of the porosities. (2) Surface recrystallization and diffusion. The combined effect of high temperature and pressure promotes atomic diffusion within the material, causing changes in the microstructure, particularly in the grains surrounding the porosities, which may undergo recrystallization or phase transformation. Thus, the gas inside the porosities is expelled, thereby effectively reducing the porosities. The results showed that the HIP diagram had good predictability for the PBF-manufactured IN718 under the HIP process.

### 5.3. Effect of HIP on Mechanical Properties

To evaluate the effect of HIP, the tensile tests were performed on specimens Case (a-3), Case (b-6), Case (c-9), and Case (d-12) at room temperature. The mechanical properties of HIP and non-HIP prints are summarized in Table 6. By closing internal properties, HIP improves the material’s overall integrity. Compared to a non-HIP print, the HIP-optimized prints exhibit higher tensile strength, fatigue resistance, and elongation due to reduced porosity and enhanced material bonding. Additionally, the print will benefit from the finer microstructure resulting from the thermal treatment, making it more comparable to wrought materials in terms of mechanical performance.

## 6. Conclusions and Future Research

### 6.1. Conclusions

Based on the above studies, some conclusions can be obtained as follows:

(1) The porosity closure condition was deduced based on the deformation analysis and the physical equation of AM materials, and the relationship between relative density, hydrostatic pressure, and yield strength was established.

(2) The relationship between temperature and yield strength was investigated by the MDM, and a porosity closure model considering temperature and pressure was proposed and verified by simulations.

(3) Ignoring the holding time effect, a HIP diagram was constructed and verified by the experiments of the PBF-manufactured IN718. The experimental results were consistent with the HIP diagram, which can be used as a reference in the actual design and optimization of HIP parameters.

### 6.2. Future Research

Future work will explore how various powder characteristics, such as shape, particle size distribution, and density, influence printing quality. By considering these factors, we aim to enhance the predictive accuracy of our model and improve its performance in capturing powder behavior during the printing process.

## Figures and Tables

**Table 1:** Element mass (%) and atomic number proportion of the simulation sample.

	Ni	Cr	Fe	Co	Cu
Element (%)	52.1	18.7	27.9	0.27	0.98
Atomic	386,270	138,524	206,704	2019	7255

**Table 2:** The specific simulation parameters.

Relative Density	Temperature (K)	Target Press (GPa)
0.956	1300	19.56
	1400	18.17
	1500	16.77
0.970	1300	21.35
	1400	19.83
	1500	18.31
0.984	1300	24.29
	1400	22.59
	1500	20.83

**Table 3:** Hatch and contour line parameters.

	No.	Laser Power (W)	Spot Diameter (μm)	Powder Thickness (μm)	Hatch Distance (μm)	Scanning Speed (mm/s)
Hatch Lines	a					500
	b					650
	c	160	130	50	100	850
	d					1100
Contour Lines						500

**Table 4:** HIP experimental schemes.

No.	Case	Holding Time (min)	Temperature (℃)	Pressure (MPa)
a	a-1	240	1050	180
	a-2		1100	180
	a-3		1150	65
b	b-4		1050	180
	b-5		1100	180
	b-6		1150	60
c	c-7		1050	180
	c-8		1100	160
	c-9		1150	55
d	d-10		1050	180
	d-11		1100	145
	d-12		1150	50

**Table 5:** Relative density before and after HIP.

Case	Before HIP			After HIP		
	$\rho_{a}$	$\rho_{core}$	ρ	$\rho_{a}$	ρ	$\rho_{amp}$
a-1	8.193	8.189	0.996	8.195	0.997	-
a-2	8.194	8.191	0.996	8.195	0.997	-
a-3	8.194	8.191	0.996	8.211	0.999	1
b-4	8.105	8.091	0.984	8.121	0.988	-
b-5	8.105	8.091	0.984	8.212	0.999	1
b-6	8.108	8.093	0.984	8.212	0.999	1
c-7	8.004	7.976	0.970	8.064	0.981	-
c-8	8.006	7.979	0.970	8.211	0.999	1
c-9	8.006	7.979	0.970	8.211	0.999	1
d-10	7.904	7.864	0.956	7.899	0.961	-
d-11	7.904	7.864	0.956	8.212	0.999	1
d-12	7.906	7.866	0.956	8.212	0.999	1

**Table 6:** Mechanical properties before and after HIP.

Case.	Before HIP			After HIP		
	Yield Strength 0.2% (MPa)	Tensile Strength (MPa)	Elongation to Failure (%)	Yield Strength 0.2% (MPa)	Tensile Strength (MPa)	Elongation to Failure (%)
a-3	673	857	4	910	1341	13
b-6	655	845	3.5	855	1237	8.5
c-9	543	756	3	873	1247	8
d-12	479	679	3	901	1299	8.5

## Data Availability

The data is unavailable due to privacy or ethical restrictions.
